# The State Medical Society

**Published:** 1877-07

**Authors:** 


### MISCELLANEOUS.

### The State Medical Society.

The Medical Society of the State of New York, convened in the Assembly Chamber Tuesday morning at eleven o'clock, and was called to order by Dr. E. R. Squibb, of Brooklyn, President of the Society. There was an attendance of over one hundred members. The session was opened with prayer by the Right Rev. Bishop Doane, of Albany.

The President then proceeded to read his opening address, in which he congratulated the members of the Society on its prosperity, and suggested for its still further advancement that the presiding officers be selected with the more single purpose of getting a good active servant of the Society who would carry out its work with impersonal energy; that the presiding officer should carry out and be responsible for the work of the Society at the meeting over which he presides; that the address which usually occupies the Wednesday evening session be discontinued, or at least the penalty of $25 for failure to deliver it, be abolished; that the Secretary's annual salary be increased to $500, or that the editorship of the Transactions be separated from the duties of the Secretary, and given to an editor; that each volume of the Transactions hereafter should have a copious index; that a list of delegates, by counties, with the time of election and expiration of service should precede the list of permanent members, and be kept up with official accuracy, so that the true character and construction of this body as a society of delegates should be more prominent, and that the Treasurer should receive compensation for the time and labor required of him by the Society.

The President further says that he has, during the year, signed a diploma for Dr. Carl Schade, of Buffalo, and also signed a petition to the Legislature for the passage of an act to provide for the sanitary inspection and supervision of common schools and school buildings in New York City. Dr. Frank H. Hamilton, of New York, had been appointed to the vacancy in the delegation to the American Medical Convention.

The President announced the following committees :

*Hygiene.*—Drs. E. V. Stoddard, of Rochester; C. R. Agnew, of New York; John T. Van Alstyne, of Columbia; James C. Hutchinson, of Rensselaer; Taber B. Reynolds, of Saratoga; E. M. Lyon, of Clinton; Edwin Hutchinson, of Oneida; J. R. Stockwell, of Oswego; T. H. Squire, of Chemung; J. G. Orton, of Broome; C. G. Pomeroy, of Wayne; Harvey Jewett, of Ontario.

*Credentials.*—Drs. Wm. Manlius Smith, of Onondaga; G. H. Blake, of Livingston; A. N. Saunders of Madison.

*Reception and Arrangement.*—Drs. E. R. Hun, Wm. H. Bailey, of Albany; A. W. Tucker, of Washington.

*Business.*—Drs. George Burr, of Broome; S. F. McFarland, of Chenango, R. H. Ward, of Rensselaer.

*Ethics.*—Drs. Wm. C. Wey, of Chemung, Austin Flint, of New York; Frederick Hyde, of Cortland.

*Nominations.*—Drs. A. Hutchinson, of Kings; P. R. H. Sawyer, of Westchester; C. E. Whitbeck, ef Albany; J. M. Rose, of Herkimer; L. A. Van Wagner, of Madison; J. H. Crittenden, of Broome; C. G. Pomfret, of Wayne; T. F. Rochester, of Erie.

A vote of thanks was then tendered the President for his address, and a recess for half an hour was taken for the purpose of organizing committees, receiving credentials, and for registration of names of permanent members.

On reconvening, the Committee on Credentials reported the members present, and Dr. Bailey of the reception committee introduced the following delegates from other State Societies : Dr. Collins, of the Massachusetts State Medical Society; Dr. Gillett, of Pennsylvania State Society, and Dr. D. A. Curry, of the New Jersey Society. Dr. Curry, when introduced, made a few witty remarks which were received with applause.

A motion that the nominating committee report a committee on President's address was adopted.

The treasurer reported the balance on hand at the last report was $287.71; received during the year, $1,384.31; disbursed during the year, $1,684.19. In the general fund the receipts were $1,922. 76, and the disbursements $2,132.31, leaving a balance due treasurer of $209.55. The report was referred to an auditing committee, with Dr. Elliott as chairman.

The Secretary, Dr. E. R. Hun, reported the number of Transactions on hand, and the report was accepted.

Dr. Bailey, from the Committee on Publication, reported that 1,500 volumes of the Transactions had been printed during the past year.

The reports of delegates to other societies were then received, and after some discussion on unimportant matters a recess was taken until three o'clock.

### AFTERNOON SESSION.

The Society was called to order by the President at 3 o'clock.

Dr. Rochester, of the nominating committee, reported as the committee on the President's inaugural address: Dr. Ellsworth Eliot, Dr. A. Van Derveer and Dr. H. Jewett, of Canandaigua.

Dr. R. W. Pease, of Syracuse read a paper on the Recent Improved Methods of Diagnosis and treatment in Urethral Surgery, with tabulated statement of results in forty-five cases. Discussed by Drs. Case of Oneonta, Wheeler of Onondaga, and others.

Dr. J. Kneeland, of South Onondaga, read a paper entitled "Two Cases of Sudden Death—Coroner's Inquests." Accepted, and referred to the publishing committee. The paper was discussed by Drs. Rochester and Graves.

The chairman of the business committee read by title a paper entitled "An Obituary Notice of James Thorn, M. D.," by R. H. Ward, M. D., of Troy. Referred to publishing committee.

Dr. A. Van Derveer, of Albany, read a paper entitled "Operation for Closure of Cleft of Hard Palate, with report of cases." The paper was discussed by Dr. Goodwilly, of New York, who presented a wax cast of a subject on which he had operated and gave an illustration of the manner in which the operation was performed, exhibiting the instruments used. Dr. Hutchinson, of Utica, also participated in the discussion. The paper was referred to the publishing committee.

Dr. William C. Wey, of Elmira, read a paper entitled "Sanitary Iuspection in Schools." Referred to publication committe.

The business committee reported a paper entitled "Hydrophobia; Rabies Canina," by John W. Greene, M. D., of New York. The president read a letter from Dr. Greene, stating that he was unable to prepare the paper on account of professional engagements.

Dr. Wey moved that Dr. Greene be requested to complete his paper within thirty days, and that it be referred to the committee on publication with power. Carried.

Dr. Greene has paid considerable attention to this subject and is thoroughly posted thereon. He is recognized by the profession as an authority on hydrophobia, and his paper will doubtless be a very valuable one.

Dr. Kneeland made some remarks on the subject, and was followed by Mrs. Dr. Mary Putnam Jacobi, and Dr. E. R. Squibb, of Brooklyn.

Dr. H. T. Hanks, of New York, read a paper entitled "The Forcible and Rapid Dilatation of the Cervical Canal, for the Cure of Antiflexion." Referred to the committee on publication.

Dr. John Ball, of Brooklyn, read a paper on "Forcible and Rapid Dilatation," etc.

Recess until eight o'clock.

### EVENING SESSION.

The Society reconvened at eight o'clock.

A communication from Dr. McIlvaine, who was appointed delegate from the Ohio State Medical Society, was read by the president. Dr. McIlvaine stated that he was not able to attend this meeting, and expressed his regrets, etc. The subject was ordered entered on the minutes.

Dr. Thomas R. Pooley of New York, read a paper entitled, "Puerperal Metastatic Iridio-Chloroiditis," which was referred to the publication committee.

The business committee then called for the paper of Dr. A. W. Tupper, entitled, "Ten Years Inside the Medical Society of the State of New York." Dr. Tupper stated that perhaps some of the statements contained in his paper might not suit some of the members and requested that the paper should be submitted to the business committee to see if it contained anything objectionable. The paper was so referred.

The business committee called for the paper of A. McLean Hamilton, M. D., entitled, "Climatic Influence in the Production of Nervous Disease." The president read a telegram from Dr. Hamilton, stating that he could not be present.

Dr. S. L. Parmalee, of Watertown read a paper entitled, "Punctured Wound of Lung, Diaphragm and Liver, with recovery," which gave rise to some discussion as to whether the liver was punctured or not. The paper was referred to the publication committee.

Dr. Joshua B. Graves, of Corning read a prper entitled, "Report of a Case of Fracture of the Base of the Skull, with recovery." Referred to publication committee. The paper was discussed by Dr. Hyde, of Cortland; Dr. Becket, of Albany; Dr. Chapman, Dr. Sawyer, of Bedford; Dr. Sherman, of St. Lawrence; Dr. Kneeland, of South Onondaga, and Dr. Kendall. The Society then adjourned to 9:30 A. M. Wednesday.

### MORNING SESSION.

The Society reconvened Wednesday morning at half-past nine o'clock, and was called to order by President Squibb.

Prayer was offered by Rev. Dr. Upson, of the Second Presbyterian church.

The minutes of the previous sessions were read and approved.

Dr. William H. Bailey, from the committee on reception and entertainment, reported a large number of visitors, who were admitted to seats in the Society.

Reports were then received from the committees on credentials, business and ethics, and after discussion were adopted.

The question of changing the date of holding the annual meeting then came up and gave rise to an extended discussion.

A motion to hold the annual meeting on the third Tuesday in January was finally adopted.

This question dispored of, the reading of papers was next in order, when Dr. Austin Flint of New York, read one on "Pneumonic Fever; grounds for considering acute pneumonia an essential fever, and not purely a local inflammation." Dr. Mary Putnam Jacobi, presented "Two cases of convulsive disorder, without convulsions." All of the papers were discussed at some length.

The subject of establishing a committee to determine the qualifications of students in medical colleges, when about to enter the profession, the services to be tendered to such colleges as may desire them, did not come up, owing to the absence of Dr. E. M. Moore, of Rochester, chairman of the special committee, which was to report on the matter.

A motion to postpone the subject for one year was made and carried.

A recess was then taken until three o'clock.

AFTERNOON SESSION.

The Society reconvened at three o'clock P. M.

Dr. Edward H. Parker, of Poughkeepsie, read a paper entitled "Heredity as a Factor in Pauperism and Crime." The paper was a practical statement of the "Tramp Nuisance" giving statistics, etc. Referred to the publication committee. The paper was discussed by Dr. Wey, of Elmira, Dr. Diamond and Dr. Kneeland.

The report of the committee on the president's address was taken from the table and amended and recommended to the committee. The report of the committee, with the exception of the portion relative to the appointment of the nominating committee was adopted.

Dr. Ellsworth Elliott, chairman of the committee on the treasurer's report, reported the report as correct, and made several recommendations relative to the collection of dues. The report was accepted and adopted.

Dr. J. W. S. Gouley, of New York, read a paper entitled "Stone in the Bladder." Referred to publication committee.

The paper was discussed by Dr. Hamilton, of New York, and Dr. Van Derveer of Albany.

Dr. George Bayles of New York, read a paper entitled "Nitrite of Amyl in Pertussis." Referred to publication committee.

D. C. H. Giberson, of Brooklyn, read a paper entitled "The Cold Bath in Scarlatina"—Clinical Notes. Referred to publication committee.

The paper was discussed by Mrs. Dr. Jacobi and Dr. Kneeland.

Dr. Alexander Hutchins, of Brooklyn, read a paper on "Jaborandi," which was referred to the publication committee.

A recess was then taken until eight o'clock.

### EVENING SESSION.

The Society reconvened at eight o'clock.

Dr. A. N. Bell, of Brooklyn, chairman of the committee on Hygiene, read an abstract of the report of the committee, and it was referred to the committee on publication.

Dr. Norman S. Snow, of Albany, read a paper entitled "Psuedo-Membranous Laryngitis:—Tracheotomy:—Relapse and Recovery." Referred to the committee on publication. The paper was discussed by Dr. Goodwilly, of New York.

President Squibb read a paper entitled "Tar Fumigations in Gangrenous Sores," written by Lewis Post, M. D., of Lodi, an honorary member of the Society. Referred to the publication committee. The paper was accompanied by a letter from Dr. Post, giving the history of a disease with which he is afflicted, supposed to have arisen from the sores of the case reported. The letter was also referred.

Dr. C. G. Pomeroy, of Newark, N. Y., read a paper on "Hydrochlorate of Ammonia—Ammonia Murias," which was referred to the publication committee. The paper was discussed by Dr. Manlius Smith.

On motion of Dr. Kendall the rules were suspended, and the report of the committee on president's address was taken from the table, but not considered.

Dr. Henry G. Piffard, of New York, read a paper entitled "Certain points relating to the Nature and Treatment of Lupus." Referred to publication committee.

The report of the committee above alluded to was then presented and was freely discussed and adopted.

Dr. Ira F. Hart's paper on "Hereditary Transmission of Disease" was read by title and referred to the committee on publication.

Adjourned to 9:30 A. M., Thursday.

